# Closed Catheter Access System Implementation in Reducing the Bloodstream Infection Rate in Low Birth Weight Preterm Infants

**DOI:** 10.3389/fped.2015.00020

**Published:** 2015-03-16

**Authors:** Lily Rundjan, Rinawati Rohsiswatmo, Tiara Nien Paramita, Chrissela Anindita Oeswadi

**Affiliations:** ^1^Neonatology Division, Child Health Department, Cipto Mangunkusumo Hospital – University of Indonesia, Jakarta, Indonesia

**Keywords:** bloodstream infection, central line-associated bloodstream infection, closed catheter access system, preterm infants, infection control practices, needleless connector

## Abstract

**Background:** Bloodstream infection (BSI) is one of the significant causes of morbidity and mortality encountered in a neonatal intensive care unit, especially in developing countries. Despite the implementation of infection control practices, such as strict hand hygiene, the BSI rate in our hospital is still high. The use of a closed catheter access system to reduce BSI related to intravascular catheter has hitherto never been evaluated in our hospital.

**Objective:** To determine the effects of closed catheter access system implementation in reducing the BSI rate in preterm neonates with low birth weight.

**Methods:** Randomized clinical trial was conducted on 60 low birth weight preterm infants hospitalized in the neonatal unit at Cipto Mangunkusumo Hospital, Jakarta, Indonesia from June to September 2013. Randomized subjects either received a closed or non-closed catheter access system. Subjects were monitored for 2 weeks for the development of BSI based on clinical signs, abnormal infection parameters, and blood culture.

**Results:** Closed catheter access system implementation gave a protective effect toward the occurrence of culture-proven BSI (relative risk 0.095, 95% CI 0.011–0.85, *p* = 0.026). Risk of culture-proven BSI in the control group was 10.545 (95% CI 1.227–90.662, *p* = 0.026). BSI occurred in 75% of neonates without risk factors of infection in the control group compared to none in the study group.

**Conclusion:** The use of a closed catheter access system reduced the BSI in low birth weight preterm infants. Choosing the right device design, proper disinfection of device, and appropriate frequency of connector change should be done simultaneously.

## Introduction

Bloodstream infection (BSI) remains a major problem which is encountered in neonatal care, especially in developing countries. Low birth weight (LBW) preterm infants are specifically more susceptible to BSI during hospitalization due to a higher requirement of vascular lines for administration of parenteral nutrition, blood products, or intravenous medications (1–3). Central line-associated bloodstream infection (CLABSI) is a major cause for longer hospital stay, increased hospital costs, and the high mortality rate of neonatal intensive care unit (NICU) patients. The CLABSI is reported to cause up to 70% of all HAI (hospital-acquired infection)-BSI in preterm infants (4, 5). The National Healthcare Safety Network (NHSN) in 2009 reported a CLABSI rate of 1.3–3.4 BSIs per 1000 catheter days, and this rate decreased in 2010 to 0.8–2.6 BSIs per 1000 catheter days (6, 7). NICUs in developing countries such as Brazil reported a higher BSI rate of 17.3 BSIs per 1000 catheter days in 2010 (8), while India noted a much higher rate of 27 BSIs per 1000 catheter days in 2011 (9). In comparison, in year 2012 the neonatal unit of Cipto Mangunkusumo Hospital (CMH) in Jakarta, Indonesia reported a BSI rate of 12.88 per 1000 catheter days, and then a slight decrease in 2013 to 10.53 BSIs per 1000 catheter days (10).

Recent studies have identified that CLABSI is largely preventable (4, 11). Several practices, such as hand washing, proper catheter insertion practices, maximal barrier precautions, cleaning the skin with chlorhexidine, and avoiding femoral site combined with timely removal of the catheter, reduced significantly the CLABSI rate from 7.7 to 1.4 per 100 catheter days in adult patients treated in intensive care unit (ICU) (12). Specifically for neonatal care, CLABSI prevention efforts must emphasize both sterile central line insertion techniques and rigorous attention to ongoing central line care and maintenance (4). The term of closed catheter access system is used interchangeably with a needleless connector (NC) system, which was previously developed for preventing needlestick injury. The Center for Disease Controls (CDC) and other research have shown that the closed catheter access system is effective in reducing CLABSI through preventing the entry of microorganisms to the vascular access (6, 7, 13–16). Although CDC (2011) recommended the needleless system to access intravenous (IV) tubing (16), it is not yet officially implemented as the standard policy in the neonatal unit of our hospital. The aim of this study was to evaluate the effects of closed catheter access system implementation in reducing the BSI rate in low birth weight preterm infants in CMH.

## Materials and Methods

### Study design and sampling method

Randomized controlled trial (RCT) was conducted in the NICU and special care nursery (SCN) of CMH from June to September 2013. All neonates born in CMH with gestational age under 37 weeks and a birth weight less than 2500 g who required vascular access (peripheral and/or central) insertion were included in the study. Informed consents were taken from the parents prior to recruitment. Patients with severe diseases who might not live more than 72 h, such as complex congenital heart disease, chromosomal abnormalities, or babies in critical conditions at birth were excluded. Sixty subjects were enrolled and randomized using a block randomization method to either receive closed catheter access system (intervention group) or non-closed system (control group). All subjects were monitored for 2 weeks for signs and symptoms of BSI due to limited funding from research grant of our hospital. Subjects showing signs of new episodes of HAI-BSI underwent laboratory assessment and blood culture tests for BSI diagnosis confirmation.

### Infection control practices in CMH

Cipto Mangunkusumo Hospital has been working in conjunction with SEA-URCHIN (South-East Asia-Using Research for Change in Hospital-Acquired Infection in Neonates) project, between Australia and four South-East Asian countries. Since June 2013, our unit has been conducting regular infection control practices training according to the materials received from the Australian team. The training includes proper hand hygiene, hospital infection control practices, central line insertion and care, and antibiotic policy. Preparation of total parenteral nutrition, solution, and medication are done under laminar airflow in the pharmacy department of CMH, and quality monitoring has been regularly conducted since 2006. Oral nystatin prophylaxis has been given since the year of 2012 for babies <1500 g and/or <32 weeks who have risk factors of getting systemic fungal infection.

### Standard procedure during the study

The peripheral venous line was indicated for the administration of fluid and medication through a short intravenous catheter. The peripheral intravenous cannula may be maintained for up to 5 days when there was no sign of local infection or phlebitis. Every vascular line insertion and change must maintain an aseptic/no touch technique. Central venous line was implemented by inserting the umbilical catheter or peripherally inserted central catheter (PICC). The umbilical catheter was maintained for no more than 5 days. A peripheral insertion central catheter was used when oral intake was inadequate for at least 7 days or if the total parenteral nutrition was required for more than 1 week. Total parenteral nutrition (TPN) administration was given through a central line, whereas medication or other fluids were given through a peripheral line to ensure minimal line breaks. The central line was removed when CLABSI was suspected and the infant’s condition did not improve after antibiotic therapy, or if there were signs of inflammation at the catheter insertion site. If the entry site was inflamed and/or there was phlebitis, each intravenous set must be changed. Every TPN bag or lipid change in the central line must be done under maximal barrier precaution. In the peripheral and central lines before the connection of the intravenous line, the catheter hub or three-way stopcock must be vigorously scrubbed using a 70% alcohol swab twice for a total of 20 s (consideration for 20 s of swabbing duration was based on SEA-URCHIN training), and the antiseptic has to be dry for 30 s. During the 2- week study period, the administration sets in both groups and the NCs in the closed system were changed every 48 h for practical purposes in accordance to the changing of the TPN bag. There were no differences in principles for line insertion and change between both the intervention and control groups. All preparations to insert peripheral and central line, sterile insertion technique and line changes, and maintenance of central line care were done according to SEA-URCHIN training.

### Closed catheter access system

**Figure 1:**
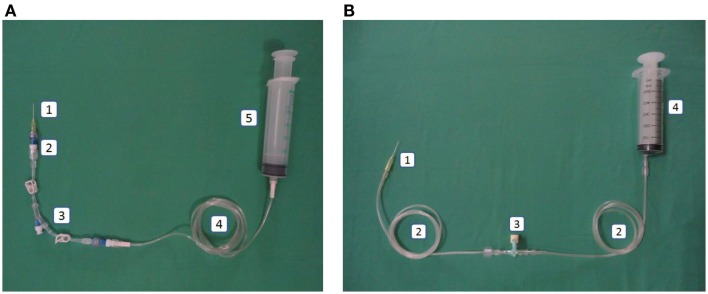
**(A)** Intervention: peripheral line in the closed catheter access system. 1. Intravascular cannula, 2. Single needleless connector, 3. Double lumen NCs with luer lock, 4. Extension tube luer lock, 5. Luer lock syringe. **(B)** Control: peripheral line in the open system. 1. Intravascular cannula, 2. Extension tube slip tip, 3. Three-way stopcock, 4. Slip tip syringe.

**Figure 2:**
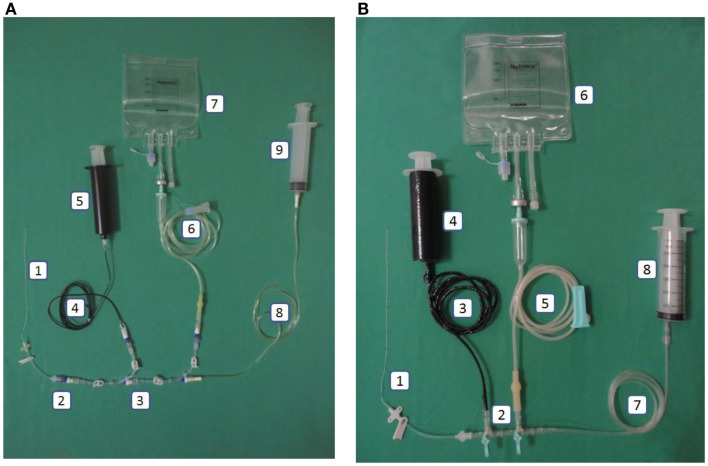
**(A)** Intervention: central line in the closed catheter access system. 1. Peripherally inserted central catheter, 2. Single needleless connector, 3. Triple lumen NCs with luer lock, 4. Extension tube luer lock (black), 5. Luer lock syringe 50mL (black), 6. Infusion set luer lock, 7. Total parenteral nutrition bag, 8. Extension tube luer lock, 9. Luer lock syringe. **(B)** Control: central line in the open system. 1. Peripherally inserted central catheter, 2. Three-way stopcock, 3. Carbon-coated extension tube slip tip, 4. Carbon-coated syringe (lipid), 5. Infusion set slip tip, 6. Total parenteral nutrition bag, 7. Extension tube slip tip, 8. Slip tip syringe.

**Figure 3:**
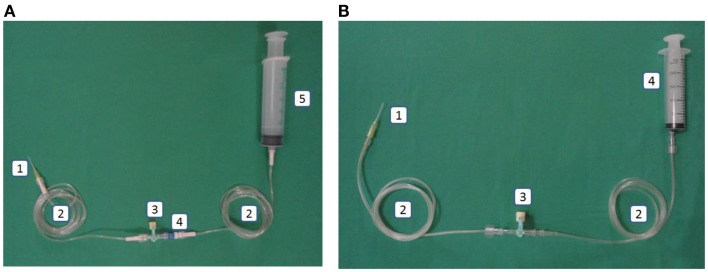
**(A)** Intervention: arterial line in the closed catheter access system. 1. Intravascular cannula, 2. Extension tube luer lock, 3. Three-way stopcock luer lock, 4. Needleless connector, 5. Heparin saline in luer lock syringe. **(B)** Control: arterial line in the open catheter access system. 1. Intravascular cannula, 2. Extension tube slip tip, 3. Three-way stopcock, 4. Heparin saline in slip tip syringe.

**Figure 4:**
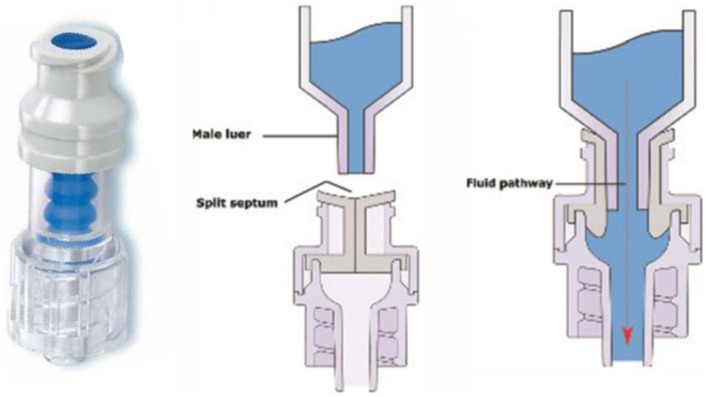
**Left: SmartSite^®^**. Center and right: fluid path mechanism (17).

The closed catheter access system was used in the peripheral venous line, central venous line, and arterial line (see Figures 1A–3A). The system was used for intravenous medication and TPN through a needleless connector and male luerlock. Administration sets consisted of an extension tube, syringe, and/or infusion set using the luerlock system to prevent the outflow of fluid through the connector (16). SmartSite^®^/Surplug^®^ (Surplug^®^ using SmartSite^®^ manufactured by ALARIS Medical Systems, is a trade mark of Terumo corporation, Japan) was the needleless connector used in our study. This NC is a split septum (SS) and mechanical valve (MV) type. When the male luer is attached, it opens the sides of the SS. Therefore, the fluid pathway is opened to allow the administration of fluid. Removing the male luer tip automatically returns to its closed position and returns the fluid pathway to its original position (see Figure 4).

### Non-closed catheter access system

This system used a slip tip port and three-way stopcock as our current practice (see Figures 1B–3B).

### Definitions

Central line-associated bloodstream infection is a form of primary BSI and not related to another site of infection (16, 18). In this study, CDC guidelines to diagnose CLABSI were used, which is laboratory-confirmed bloodstream infection (LCBI) in patients with a central line placed for >2 days prior to the occurrence of clinical symptoms (19). Presumed sepsis was defined as the presence of clinical signs of sepsis in the normal full blood count, C-reactive protein (CRP) and immature to total neutrophil (IT) ratio, and absence in micro-organism growth in blood culture. Probable sepsis was defined as a neonate suspected clinically to have sepsis with CRP >6 mg/dL and at least one of the following rapid diagnostic tests recorded positive: thrombocytopenia (<150,000/μL), IT ratio >0.2, white blood count <5000/μL or >30,000/μL (first week)/>15,000/μL (after the second week), the blood culture was negative. Culture-proven BSI was defined as a neonate showing clinical signs of sepsis with an abnormal laboratory result as above and a positive blood culture (20).

Signs and symptoms of BSI were defined at least one of the following signs or symptoms: fever (>38°C), hypothermia (36°C), apnea, or bradycardia (16).

Maternal risk factors were defined as the presence of otherwise unexplained maternal fever greater than 38^°^C with at least two of the following additional clinical findings: maternal tachycardia (>100 bpm), fetal tachycardia (>160 bpm), elevated maternal white blood cell count (>15,000 cells/μL), uterine tenderness, and foul smelling amniotic fluid (21).

### Statistical analysis

All the statistical analysis were conducted using SPSS version 22.0 (Chicago, IL, USA). Analysis was conducted to compare the two groups with respect to gender, gestational age, birthweight, maternal risk factor of infection, clinical signs of infection, and mortality rate in 2 weeks. Chi-square and Fisher’s exact tests were used. *p* Value of <0.05 was considered as statistically significant. This study was approved by the Ethics Committee of the Faculty of Medicine, University of Indonesia.

## Results

The study recruited a total of 60 low birth weight infants, consisting of 30 infants in each group. No surgical baby was recruited. Both groups did not differ significantly in terms of gender, gestational age, maternal risk factors for infection, and mortality rate in 2 weeks (Table 1).

**Table 1:** **Baseline characteristics of subjects**.

	Intervention (*n* = 30)	Control (*n* = 30)
Sex, *n* (%)
Male	14 (46.7%)	13 (43.3%)
Female	16 (53.3%)	17 (56.7%)
Gestational age, mean (SD) weeks	32.4 (SD 1.81)	32.6 (SD 2.1)
Birth weight, mean (SD) grams	1582 (SD 283.4)	1648.17 (SD 343.23)
Maternal risk factors of infection, *n* (%)
Yes	24 (80%)	26 (86.7%)
No	6 (20%)	4 (13.3%)
Death, *n* (%)	1 (3.33%)	1 (3.33%)

*SD, standard deviation*.

The incidence of culture-proven BSI in the intervention group was significantly lower than that of the control group (3.3 vs. 26.7%, *p* = 0.026, Table 2). The most common organisms in both groups were *Acinetobacter baumanii* (*n* = 5), followed by *Panthoea* sp. (*n* = 2), and *Candida parapsilosis* (*n* = 2).

**Table 2:** **Culture-proven BSI occurrence in both groups**.

	No BSI	BSI	Total	*p*
Intervention	29 (96.7%)	1 (3.3%)	30	0.026*
Control	22 (73.3%)	8 (26.7%)	30	
Total	51	9	60	

*Chi-square test.

Chi-square statistical test results showed that a closed catheter access system implementation gave a protective effect toward culture-proven BSI (relative risk 0.095, 95% CI 0.011–0.85, *p* = 0.026). The control group had a 10.545 times higher risk of BSI compared to the study group (95% CI 1.227–90.662, *p* = 0.026, Table 2). The closed catheter access system also prevented the occurrence of presumed BSI (clinical signs of infection) with relative risk of 0.203 (95% CI 0.066–0.620, *p* = 0.004, Table 3). A similar result was also found in the probable BSI (clinical signs of infection followed by abnormal infection parameters) with relative risk of 0.203 (95% CI 0.066–0.62, *p* = 0.008, Table 3).

**Table 3:** **Presumed and probable BSI occurrence in both groups**.

	Not BSI	BSI	Total	*p*
**Presumed BSI**
Intervention	17 (56.7%)	13 (43.3%)	30	0.004*
Control	7 (23.3%)	23 (76.7%)	30	
**Probable BSI**
Intervention	23 (76.7%)	7 (23.3%)	30	0.008*
Control	12 (40.0%)	18 (60.0%)	30	

*Chi-square test.

During 2-week of observation, both groups showed a difference in average sepsis episodes. The repeated culture-proven BSI incidence increased significantly in the control group. (*p* = 0.038). Two infants in the control group had two sepsis episodes and one infant had three sepsis episodes compared to no multiple sepsis episodes in the study group.

Among the 10 infants without maternal risk factors of infection (six in the study group and four in the control group), BSI occurred in 75% of infants in the non-closed catheter access system group compared to none in the study group (Table 4).

**Table 4:** **BSI in infants without previous infection**.

	Not Sepsis	Sepsis	Total	*p*
Intervention	6 (100%)	0 (0%)	6 (100%)	0.033*
Control	1 (25%)	3 (75%)	4 (100%)	

*Fisher’s exact test.

## Discussion

Low birth weight infants are vulnerable to BSI due to their immature immune system, prolonged hospital stay, and high exposure to invasive interventions (22–24). One of the most common interventions is vascular line insertion to administer medications and total parenteral nutrition. Several studies have shown that peripheral and central vein catheter insertion have similar risks for the occurrence of sepsis (22, 25).

A stopcock has been used in global medical practices, including in CMH. However, the stopcock is a potential portal of entry for microorganisms into vascular access catheters and IV fluids (16). On the other hand, the needleless intravascular catheter system was initially developed for the purpose to prevent the incidence of needlestick injury to healthcare personnel and the system was found to be more effective in reducing the risk for transmission of bloodborne infections compared to the stopcock (16, 26). Guidelines for the Prevention of Intravascular Catheter-Related Infections issued by CDC in 2011 recommended the use of the needleless intravascular catheter system with the key points including the needleless components and administration sets are changed no more frequently than every 72 h. All components used must be compatible to minimize leaks and breaks in the system, scrubbing the access port with an appropriate antiseptic, also accessing the port only with sterile devices (16). During the 2-week study period, change of the administration set and the NCs was conducted every 48 h for practical purposes in accordance to changing the TPN bag. According to CDC recommendation, the policy to change the connectors every 48 h was unnecessary (16). The study results showed that the use of the closed catheter access system gave a protective effect toward culture-proven BSI; this was similar to other studies (13–15, 22).

Furthermore, another important factor which contributes to zero CRBSI is appropriate device design (27). There are several types of needleless connectors on the market, which have different mechanisms of access, access portal, flow path, and the fluid displacement (17, 27). This study used Smartsite^®^/Surplug^®^ with a SS as its access portal and a MV with a negative fluid displacement (NFD). The main reason to choose Smartsite^®^/Surplug^®^ in this study is due to its comparatively lower price. There are four NCs available in Indonesia, three NCs are NFD type, and one is neutral fluid displacement (NeuFD) type. Other NCs’ prices are two- to three-fold more expensive than Smartsite^®^/Surplug^®^. CDC recommended the use of a SS valve as an access portal over some MVs due to the increased risk of infection with the MVs (16). While the Society for Healthcare Epidemiology of America (SHEA) and Infectious Diseases Society of America (IDSA) advised not to use positive fluid displacement (PFD) mechanical valve-needleless connector (MV-NCs) routinely due to high infection rate, Jarvis et al. in 2009 recommended the avoidance should also include NFD MV-NCs as well (27–29). However, a review from Canadian Agency for Drugs and Technologies in Health (CADTH) showed there were no differences between NFD and PFD type in terms of catheter-related infections based on three RCTs and one of the observational studies showed that the PFD-MV type was associated with more CRBSI than the NFD type (30). In addition, the Smartsite^®^ had been reported to have less bacterial transfer than other connector designs (27). The results of this research also showed that the use of the closed catheter access system gave a protective effect toward culture-proven BSI, regardless of the use of NFD.

Most MV-NCs are opaque, making it difficult to see inside to determine if there is any residual of blood, nutritional fluid remains, or there is debris remaining inside (17, 28). Ryder reported there was no significant difference of bacterial transfer and biofilm formation on the intraluminal catheter surface between Clearlink^®^ (clear device) and Smartsite^®^ (31). Besides that, the external surface of NC maybe associated with infection risk. The needleless connector may have some surface configurations, such as: flat, angled post in the center, or irregular form, and also with or without a new sterile end cap. The more complicated designs made if difficult to swab the surface adequately before use (17). The Smartsite^®^ has a flat and easy swab surface, so that the health personnel would easily be able to clean the surface before manipulation.

Esteve et al. (32) reported a different result about Smartsite^®^ that it did not reduce the incidence of catheter-related bacteremia in adult patients of the ICU. In their study, there was a different treatment between two groups; NCs were disinfected with 0.5% chlorhexidine digluconate solution, while there was no swabbing on the three-way stopcocks (32). Another prospective study with a different result was Salgado et al. (33) in a medical facility for adult patients with long-term hospitalization. This study concluded that the use of NFD-NC switched from a needleless split-septum device (NSSD) could increase CRBSI. In their study, the connector was changed every 96 h, unless there were blood products or parenteral nutritions administered and only swabbed 3–5 s before manipulation (33). Method differences between Salgado’s study and this study may give different results. Ryder et al. (31) compared the bacterial transfer rate of eight NCs. This study inoculated bacteria on the surface of NC twice a day and dried for 30 min before attaching to the catheter. The connector-catheter sets were flushed with sterile normal saline, collected, then plated. The result showed that NeuFD NC had the smallest mean log densities (LD) surface inoculation compared with others (31). However, until now no studies have been found which compare the NCs type in newborn infants.

The implementation of the proper aseptic device management in this study is ensured. The time duration of antiseptic swabbing is not mentioned in the CDC recommendation. This studies’ consideration for 20 s of swabbing duration was based on SEA-URCHIN training. A study conducted by Rupp et al. (34) showed no significant difference in microbial contamination rates among 5-, 10-, 15-, and 30-s of disinfection time. The study concluded that a 5 s scrub with 70% isopropyl alcohol gives adequate disinfection of a split-septum intravascular catheter. Nevertheless, *in vitro* assessment by Rupp et al. reported that the connector valves should be disinfected ≥10 s to sterilize very heavy contamination (10 8 CFU per connector diaphragm) (34). Similarly, other studies have recommended routine disinfection of catheter connector valves for 15 s or longer (6, 35, 36). The choice of antiseptic in CDC is chlorhexidine, povidone iodine, an iodophor, and 70% alcohol (16). Kaler et al. in 2007 reported that alcohol alone or alcohol based chlorhexidine is equally effective in sterilizing NC with 15 s swabbing duration, regardless of whether NCs are negative, positive, or neutral fluid displacement type (37). The decision to use 70% isopropyl alcohol in the CMH unit is because it is broadly effective against a wide variety of bacterial and fungal pathogens and also is inexpensive and easily available (34, 37).

The limitations in this study are the limited number of subjects without risk factors of infection and the short follow-up time. In this study, it was shown that the majority of subjects (75%/three out of four infants) without risk factors of infection in the control group had sepsis during 2 weeks of observation, while none was infected in the study group. However, this percentage may not represent the same probability for all subjects without risk factors of infection. To date, there are still a small number of studies regarding the use of SS-negative fluid displacement design globally, and none in Indonesia. This study is the first internal Indonesian study regarding the use of the needleless catheter system. Another study conducted with a bigger number of subjects without risk factors of infection especially with same device design (NFD-NCs) is encouraged.

In conclusion, our study showed that the NCs/closed catheter system reduced the CRBSI rate compared to the non-closed catheter access system. Furthermore, in order to maintain lower CRBSI rate not only the right choice of device design, but also the disinfection of access port and frequency of administration sets should be done properly and at the same time.

The encouraging results of this study hopefully will add to the knowledge of medical providers and policy makers regarding the closed catheter access system as the possible intervention to reduce BSI rate. Although the cost spending for the use of NCs in this study, advantages gained by using the system outweigh the potential higher cost due to longer hospital stay in case of BSI occurrence.

## Conflict of Interest Statement

This study was funded by a research grant from Cipto Mangunkusumo Hospital. The authors declare no conflict of interest with the manufacturers and distributors (Terumo).
